# Predicting photoactivity in dithienylethene crystalline solids

**DOI:** 10.1107/S2052252523008990

**Published:** 2023-10-17

**Authors:** Kristin M. Hutchins

**Affiliations:** a Department of Chemistry, University of Missouri, 601 S. College Ave, Columbia, Missouri 65211, USA

**Keywords:** crystal engineering, di­aryl­ethene, photoswitch, crystal landscape

## Abstract

This commentary discusses the design of stimuli-responsive materials, specifically, light-responsive dithienylethene-based compounds. Recent progress in predicting photoactivity using a combination of theory and crystal structure landscape experiments is highlighted.

Stimuli-responsive materials are a unique class of compounds that undergo a change in response to an applied stimulus. Common stimuli include light, temperature, force and chemical, while the response of the material to such stimuli can vary widely, including changes in shape, color and stiffness, among others (Li, Iscen *et al.*, 2020 ▸; Roy *et al.*, 2010 ▸; Yan *et al.*, 2022 ▸; Yang *et al.*, 2023 ▸). Materials that respond to stimuli have attracted attention because they can provide information about the environment around them. For example, materials that exhibit color changes can be used for *anti*-counterfeiting, changing lenses, or to indicate applied force (Li, Huang *et al.*, 2020 ▸; Malic *et al.*, 2010 ▸; Qian *et al.*, 2021 ▸). At the molecular level, these changes typically arise from a change in structure, which is initiated by the stimulus.

Photoswitchable materials contain a molecule that is capable of changing its structure in response to the application of light as a stimulus. For solid or crystalline phase materials, there are typically requirements that need to be met for the molecule to be photoactive and undergo the structural change because motion in solids is more restricted. Dithienylethenes are one class of photoswitchable materials that can be converted between an open and closed form, depending on exposure to certain wavelengths of light [Fig. 1 ▸(*a*)]. Dithienylethenes are promising candidates for incorporation into organic photoswitches because they react in the solid state, both forms are thermally stable, and they are resistant to fatigue (Herder *et al.*, 2015 ▸; Kitagawa *et al.*, 2013 ▸; Kobatake *et al.*, 2000 ▸; Shibata *et al.*, 2002 ▸). For a dithienylethene to be considered potentially photoactive in the solid state, the molecule should adopt an antiparallel geometry and the two active carbon atoms should have an interatomic distance of less than 4.2 Å (Kobatake *et al.*, 2002 ▸). However, the conformational flexibility of dithienyl­ethenes (via free rotation of thio­phene rings) creates challenges in designing and achieving conformations that are photoactive in the solid state. In this issue of **IUCrJ** , Benedict and coworkers take on this challenge by preparing several solid-state forms of a dithienylethene derivative and developing a facile and robust analysis method that can be used to predict photoreactivity in the system (Mitchell *et al.*, 2023 ▸). By pairing experimental crystal structure data with calculations, the described method provides an easy way to relate structure to property. Importantly, the method could be applied more broadly to other dithienylethene systems to predict solid-state photoactivity.

Crystal engineering, the rational design of functional molecular solids (Desiraju, 1995 ▸; Etter, 1990 ▸; Metrangolo *et al.*, 2005 ▸; Moulton & Zaworotko, 2001 ▸; Schmidt, 1971 ▸), offers a strategy for obtaining solid-state structures that are expected to be photoactive. However, if there are few structures known, or if suitable design strategies have not been developed for a given class of molecules, ‘engineering’ the solid to exhibit a desired structure, and thus, function, becomes more challenging. Therefore, the development of structural landscapes for such classes of molecules is useful (Desiraju, 2017 ▸, 2021 ▸). By determining several structures and taking each structure as a data point on the landscape, structure–property relationships can be developed.

In the article by Benedict and coworkers, the crystal structure landscape of the molecule (*Z*)-1,2-bis­(2-methyl-5-(pyridin-4-yl)thio­phen-3-yl)-1,2-di­phenyl­ethene, which is abbreviated ‘DTE’ is investigated [Fig. 1 ▸(*b*), left]. By incorporating DTE into a variety of solids, and, thus, obtaining several data points, the authors aim to gain a broad understanding of behavior and develop the structural landscape. Specifically, the authors investigate an array of compound types including the parent compound, hydrogen-bonded cocrystals and coordination compounds. The parent compound, DTE, is polymorphic and two structures were obtained. Through cocrystallization with multitopic carb­oxy­lic acids, four hydrogen-bonded cocrystals were prepared. Six coordination polymers were synthesized with DTE as a ligand. Finally, seven solids were obtained from metal-organic framework syntheses using zinc, DTE and multitopic carb­oxy­lic acids. In total, 19 structures were used to form the basis of the structural landscape for this pyridyl-functionalized dithienylethene derivative.

Using established requirements for photoactivity in dithienyl­ethene compounds (*i.e.* antiparallel geometry and interatomic distance less than 4.2 Å), the authors visually examined each DTE molecule within each solid-state structure. Within the 19 solids, 17 contained photoinactive DTE molecules and two contained photoactive DTE molecules. Considering the broad class of compounds prepared in the study, the work shows that obtaining photoactive conformations in the solid state is not trivial.

The authors conducted relaxed potential energy surface scans using a geometry-optimized DTE molecule, and the torsion angle [Fig. 1 ▸(*b*), blue atoms] was rotated through ±1° increments to create a 360° analysis of the molecule with corresponding energies for each position. These calculations afforded four energetic minima overall, which correspond to four unique conformations, two antiparallel and two parallel. Since an antiparallel arrangement is required for photoactivity, the authors looked at the distance between the active carbons in the two minimum energy antiparallel conformations. One conformation had an interatomic distance of 3.45 Å, while the second was 5.19 Å; thus, only the former conformation would be expected to be photoactive. The authors mention that visual inspection of conformers can be time-consuming and somewhat subjective; thus, they sought to develop a simple method to determine conformer type and photoactivity potential unambiguously.

The first parameter they define is *D*_active_, which is simply the measured distance between the active carbons, and the distance should be less than 4.2 Å to be photoactive. The second parameter they define is *D*_Me–Me_, which is the distance between the two methyl groups on the thio­phene rings [Fig. 1 ▸(*b*), right]. *D*_Me–Me_ will give values that are invariable with respect to structure inversion, *i.e.* enantiomers would afford identical values. Plotting the values of *D*_active_ versus *D*_Me–Me_ using the calculated structures affords a broken ellipse shape, with three distinct areas that are populated [Fig. 1 ▸(*c*)]. The regions include: (I) antiparallel conformations with *D*_active_ < 4.2 Å, (II) antiparallel conformations with *D*_active_ > 4.2 Å and (III) parallel conformations. Moreover, when the experimentally obtained values from the 19 unique X-ray crystal structures are added to the plot, they also fall into the same three regions. The two synthesized DTE solids that were experimentally photoactive fall into region I, which is indeed the expected photoactive region [Fig. 1 ▸(*d*)].

This work demonstrates the power of experiment and theory conducted in tandem for predicting properties in crystalline materials. The *D*_active_–*D*_Me–Me_ analysis provides a straightforward way to predict photoactivity using only two distance measurements. Rapid and effective tools, such as the analysis method described, are valuable to the crystal engineering community. First, such tools are useful to understand why a given structure affords a property. Second, and where the tool shows effectiveness, is when it can be used with crystal engineering strategies to design materials that exhibit desired solid-state conformations and corresponding properties. Further application of this tool to DTE and other dithienyl­ethene derivatives will aid in achieving photoactive conformations (and photoswitching) in the solid state.

## Figures and Tables

**Figure 1:**
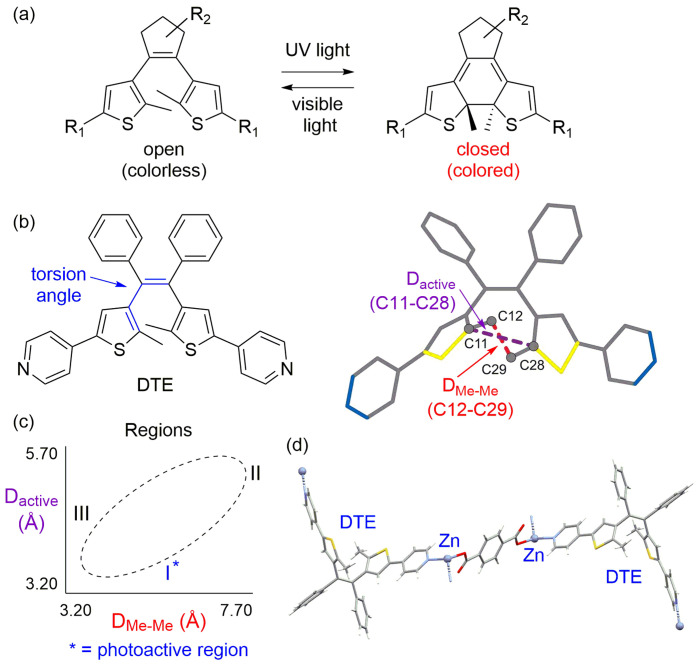
(*a*) General dithienylethene structure in open and closed form. (*b*) Pyridyl-functionalized dithienylethene derivative (DTE) highlighting atoms used in torsion angle measurements (blue), the distance between active carbons (*D*_active_) and distance between methyl groups (*D*_Me–Me_) (Mitchell *et al.*, 2023 ▸). (*c*) Plot of *D*_active_ versus *D*_Me–Me_ showing locations of the three distinct and populated regions. (*d*) One of the two photoactive compounds obtained with DTE.
